# MT-Toolbox: improved amplicon sequencing using molecule tags

**DOI:** 10.1186/1471-2105-15-284

**Published:** 2014-08-22

**Authors:** Scott M Yourstone, Derek S Lundberg, Jeffery L Dangl, Corbin D Jones

**Affiliations:** Curriculum in Bioinformatics and Computational Biology, University of North Carolina, Chapel Hill, 27599 USA; Department of Biology, University of North Carolina, Chapel Hill, 27599 USA; Curriculum in Genetics and Molecular Biology, University of North Carolina, Chapel Hill, 27599 USA; Department of Microbiology and Immunology, University of North Carolina, Chapel Hill, 27599 USA; Carolina Center for Genomic Sciences, University of North Carolina, Chapel Hill, 27599 USA; Howard Hughes Medical Institute, University of North Carolina, 400 Jones Bridge Road, Chevy Chase, MD 20815-6789 USA

**Keywords:** Amplicon, Sequencing, Molecule tagging, Sequencing error, PCR Bias

## Abstract

**Background:**

Short oligonucleotides can be used as markers to tag and track DNA sequences. For example, barcoding techniques (*i.e*. Multiplex Identifiers or Indexing) use short oligonucleotides to distinguish between reads from different DNA samples pooled for high-throughput sequencing. A similar technique called molecule tagging uses the same principles but is applied to individual DNA template molecules. Each template molecule is tagged with a unique oligonucleotide prior to polymerase chain reaction. The resulting amplicon sequences can be traced back to their original templates by their oligonucleotide tag. Consensus building from sequences sharing the same tag enables inference of original template molecules thereby reducing effects of sequencing error and polymerase chain reaction bias. Several independent groups have developed similar protocols for molecule tagging; however, user-friendly software for build consensus sequences from molecule tagged reads is not readily available or is highly specific for a particular protocol.

**Results:**

MT-Toolbox recognizes oligonucleotide tags in amplicons and infers the correct template sequence. On a set of molecule tagged test reads, MT-Toolbox generates sequences having on average 0.00047 errors per base. MT-Toolbox includes a graphical user interface, command line interface, and options for speed and accuracy maximization. It can be run in serial on a standard personal computer or in parallel on a Load Sharing Facility based cluster system. An optional plugin provides features for common 16S metagenome profiling analysis such as chimera filtering, building operational taxonomic units, contaminant removal, and taxonomy assignments.

**Conclusions:**

MT-Toolbox provides an accessible, user-friendly environment for analysis of molecule tagged reads thereby reducing technical errors and polymerase chain reaction bias. These improvements reduce noise and allow for greater precision in single amplicon sequencing experiments.

**Electronic supplementary material:**

The online version of this article (doi:10.1186/1471-2105-15-284) contains supplementary material, which is available to authorized users.

## Background

High-throughput sequencing has revolutionized biological science and biomedical research. However, erroneous base calls reduce the information value of each sequence, and polymerase chain reaction (PCR) bias leads to inaccurate quantification of sequences. To address these limitations several methods have been developed where randomly generated oligonucleotides are used as a molecule tag (MT). Molecule tagging should not be confused with barcoding (*i.e*. Multiplex Identifiers or Indexing) where short oligonucleotides are used to tag individual samples, which are then pooled and simultaneously sequenced. The resulting reads are then informatically sorted by the sample barcode. Molecule tagging is a similar idea where unique tags are attached to individual DNA template molecules within a sample prior to exponential PCR amplification (Additional file 1: Figure S1.A). After PCR and sequencing, reads sharing the same MT likely originated from the same template molecule, meaning that discrepancies among these reads can be attributed to technical error. Forming consensus sequences (ConSeqs) from reads with the same MT corrects these errors. Additionally, any preferential PCR amplification biases are mitigated because ConSeqs represent the original population of templates [1–3].

Molecule tagging is useful for a variety of applications. For instance, Kinde *et al*. [4] used molecule tagging to test polymerase fidelity, accuracy of *in vitro* synthesized oligonucleotides, and prevalence of mutations in nuclear and mitochondrial genomes of normal cells. Jabara *et al*. [5] used molecule tagging to detect and quantify single nucleotide polymorphisms (SNPs) in the HIV—1 protease gene in complex viral populations. Kivioja *et al*. [1] showed how molecule tagging improves quantification of mRNA sequencing experiments. Faith *et al*. [6] used a molecule tagging method called Low-Error Amplicon Sequencing (LEA-Seq) for metagenomic 16S gut profiling and observed a substantial reduction in the observed microbial community complexity due to the elimination of spurious sequences. Lundberg *et al*. [7] saw a similar reduction in 16S microbial complexity when profiling microbially diverse bulk soil samples. In each of these studies, molecule tagging allowed greater confidence in the amplicon sequences and their quantification.

Despite extensive efforts developing and using these error-reducing protocols, software for building ConSeqs in the previously cited projects [4–6] is not readily available or is highly specific for a particular application (*e.g*. [5]). For example, LEA-Seq scripts can only be run on a small number of 16S amplicons sequenced using paired-end 108 bp Illumina reads with a single 12-20 bp molecule tag. This specificity makes LEA-Seq scripts less practical for most amplicon experiments that could benefit from molecule tagging. Consequently, we developed MT-Toolbox (Molecule Tag Toolbox), a flexible and user-friendly software package to generate ConSeqs from molecule tagged reads produced from several different MT protocols.

## Implementation

**Figure 1:**
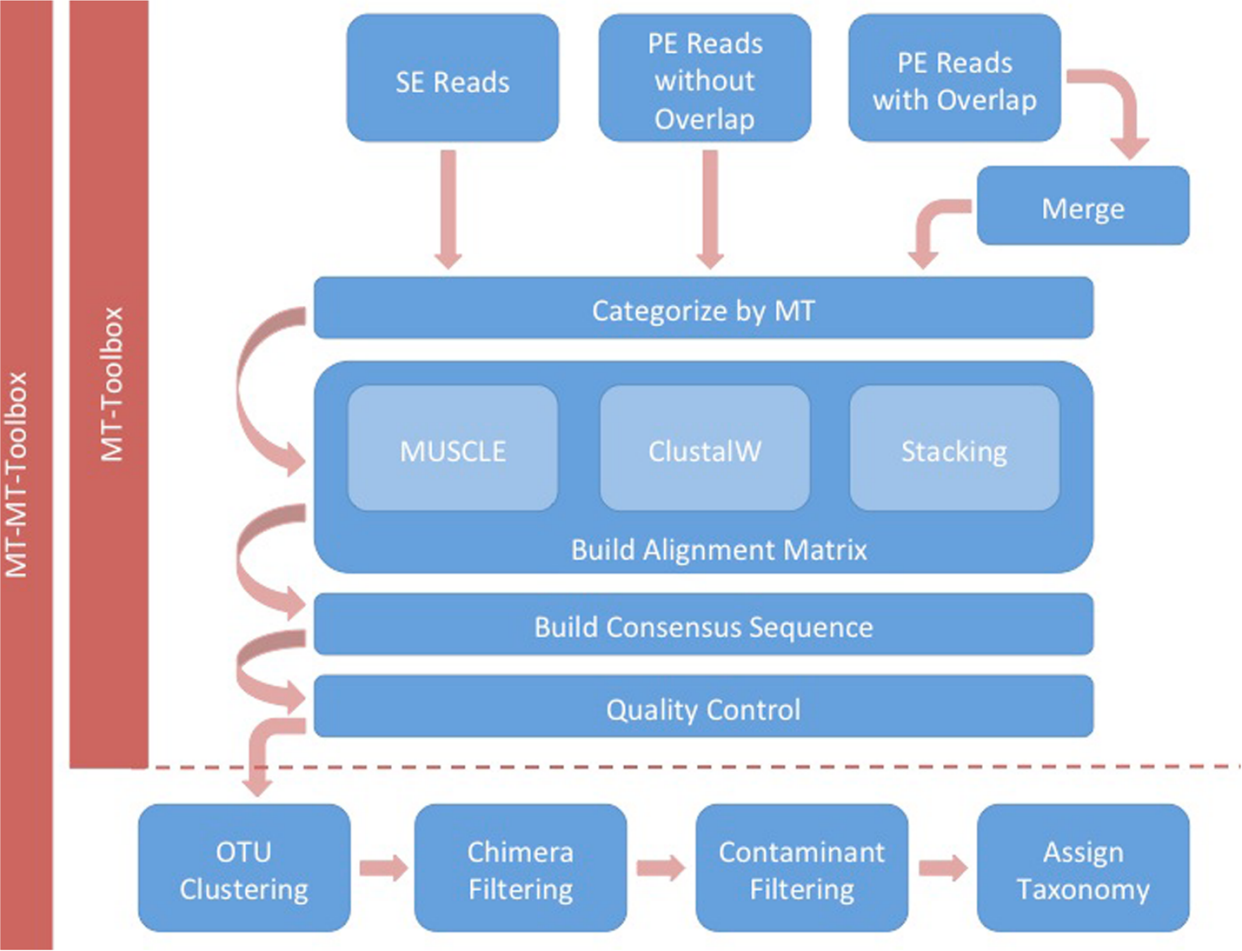
**MT-Toolbox overview.** Single-end or paired-end (overlapping or non-overlapping) reads can be input into MT-Toolbox. Overlapping paired-end reads are merged after which all reads, regardless of their type, are categorized by their MT. Next a square alignment matrix is created for each MT category using either an MSA algorithm (e.g. ClustalW, MUSCLE) or by read stacking. From these matrices, ConSeqs are built and quality control measures remove low-quality ConSeqs. When using the MT-MT-Toolbox plugin, ConSeqs are subjected to traditional 16S profiling analyses including OTU clustering, chimera filtering, contaminant filtering, and assigning taxonomy.

The primary purpose of MT-Toolbox is to categorize reads by MT and build ConSeqs (Figure 1). MT-Toolbox can categorize and correct single-end (SE), overlapping paired-end (PE), and non-overlapping PE reads. With overlapping PE reads, a preprocessing step runs FLASH [8] to merge corresponding PE reads into a single sequence. Regular expressions, a common pattern matching technique, are used to identify the expected regions (*e.g*. MT, primer, amplicon) of each read (Additional file 1: Figure S1.B-D). Reads matching the regular expression are then categorized by their MT.

The first step in identifying and correcting errors among reads having the same MT is building a square alignment matrix, **M** (Additional file 1: Note S.1). This matrix is described as each row, *r*, representing a read and each column, *c*, containing a single base from read *r* at position *c*. The number of rows (i.e. number of reads) in **M** is referred to as the MT depth and is an important parameter in evaluating the accuracy of final ConSeqs. Multiple sequence alignment (MSA) programs such as ClustalW [9] or MUSCLE [10] can be used to generate such a matrix. However, computational overhead from operations like file input/output associated with these programs requires a substantial amount of time (Additional file 1: Figure S2). Alternatively, **M** can be created without using an MSA program by simply stacking reads. Because reads in an MT category are likely to originate from the same template molecule, they are likely to have uniform lengths (Additional file 1: Figure S3). Furthermore, Illumina sequences rarely incorporate insertions or deletions into sequenced reads (Additional file 1: Figure S4). In the rare case where reads in a single MT category differ in lengths, reads are clustered by length and only reads from the largest cluster are used to build the ConSeq. If multiple clusters are equally represented as the largest cluster, one of them is arbitrarily chosen to build the ConSeq. Comparisons of ConSeqs generated by ClustalW, MUSCLE, and the read stacking method show that ConSeqs derived from stacked reads are only slightly less accurate (Additional file 1: Figure S5), and reduce runtime by ~54%. While options for using either ClustalW or MUSCLE are available in MT-Toolbox, the default is to stack reads.

From **M** a consensus sequence can be built by choosing the mode base in each column (Additional file 1: Figure S6). The quality score of the consensus base is set to be the mean of the original quality values of the mode base. Ties are resolved by choosing the base with the highest average quality score. If a tie cannot be resolved using quality scores, an IUPAC encoding is used as the consensus base. Using quality score information provides a major advantage because ConSeqs can be generated from MTs represented by only two reads thereby keeping a larger proportion of reads. This is especially important for samples with high amplicon population diversity because it captures a larger fraction of the population. This is an improvement over LEA-Seq, which cannot build ConSeqs from MTs having a depth of two where the reads are not identical. Furthermore, other ConSeq building software (*e.g*. [5]) use only sequence information to build consensus sequence and thus are only able to generate ConSeqs from MTs having a depth greater than two reads.

The primary output file contains ConSeqs and corresponding quality scores in FASTQ format. A second FASTQ file contains single read categories (SRCs; MTs with only one raw read) that can optionally be included in downstream analysis (Additional file 1: Note S.2). However, SRCs retain all technical errors associated with sequencing and PCR because no consensus sequence can be generated from a single read. Quality control parameters (Additional file 1: Note S.3) allow filtering of low quality ConSeqs and SRCs, ConSeqs with low depth, and ConSeqs where a single MT tags two different templates by chance (*i.e*. the ‘*birthday paradox*’) [11, 12] (Additional file 1: Note S.4, Figures S7 and S8).

MT-Toolbox also includes the following features: 1) jobs can be started via a graphical user interface (GUI) or command line interface (Additional file 1: Figure S9), 2) an additional plugin provides features for 16S microbial profiling, namely—building operational taxonomic units (OTUs), assigning OTU taxonomy, and removing contaminant OTUs (Figure 1, Additional file 1: Note S.5) using the MeTagenomics plugin (MT-MT-Toolbox, Additional file 2), 3) the BioUtils library (Additional file 1: Note S.6, Figure S10; Additional file 3), digital normalization [13] parameters (Additional file 1: Note S.7), and optional Load Sharing Facility (LSF) based cluster parallelization (Additional file 1: Note S.8) reduce runtime and memory requirements, and 4) an MT-Toolbox website provides descriptions, tutorials, installation instructions, updates, and other important documentation [14].

MT-Toolbox is implemented as a suite of object-oriented Perl modules and scripts (Additional file 4). It has been successfully tested on Perl versions 5.8.8, 5.8.9, and 5.12.3. Several external Perl modules are required, and can be easily downloaded and installed via a simple build command before building and installing MT-Toolbox. The GUI was built using the Perl/Tk library and requires an X Window System. MT-Toolbox also uses gnuplot 4.4 for generating simple summary graphs. The optional MT-MT-Toolbox plugin allows for standard 16S microbial profiling analysis. MT-MT-Toolbox requires USEARCH v7.0.1090 [15] or greater for OTU clustering and chimera filtering, the RDP Classifier [16] as implemented in QIIME [17] for OTU taxonomy classification, and BLAST + 2.2.25 [18] for contaminant sequence removal.

## Results

**Figure 2:**
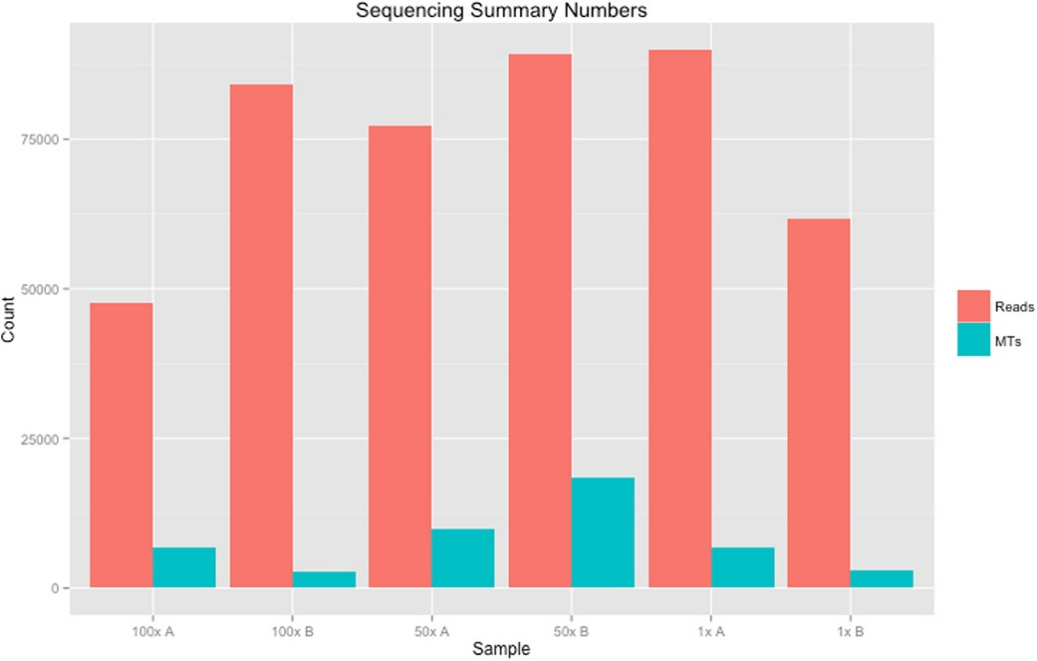
**Read and MT counts per sample.** Here reads are defined as the number of raw reads that can be categorized (“categorizable”). In other words, the read matches the expected regular expression pattern for merged reads (Additional file 1: Figure S1.C). MT-Toolbox assigns each categorizable read to an MT category. MT counts are the number of MT categories (*i.e*. number of originally tagged DNA templates). The sum total of reads in each MT category equals the number of categorizable reads.

To show the utility of ConSeqs generated by MT-Toolbox, we used data from [7] which consists of a clonal plasmid containing a known 16S gene. From this single clonal plasmid, separate DNA samples were created by performing two replicate dilutions of 1x, 50x, or 100x, for a total of six samples. Each sample was molecule tagged and PCR amplified. It is important to note that each sample should contain just one “real” amplicon matching the original 16S amplicon in the clonal plasmid. Samples were barcoded, pooled, and sequenced on an Illumina MiSeq platform using standard 2 × 250 bp protocols. After demultiplexing samples, ConSeqs were generated using MT-Toolbox (Figure 2).

**Figure 3:**
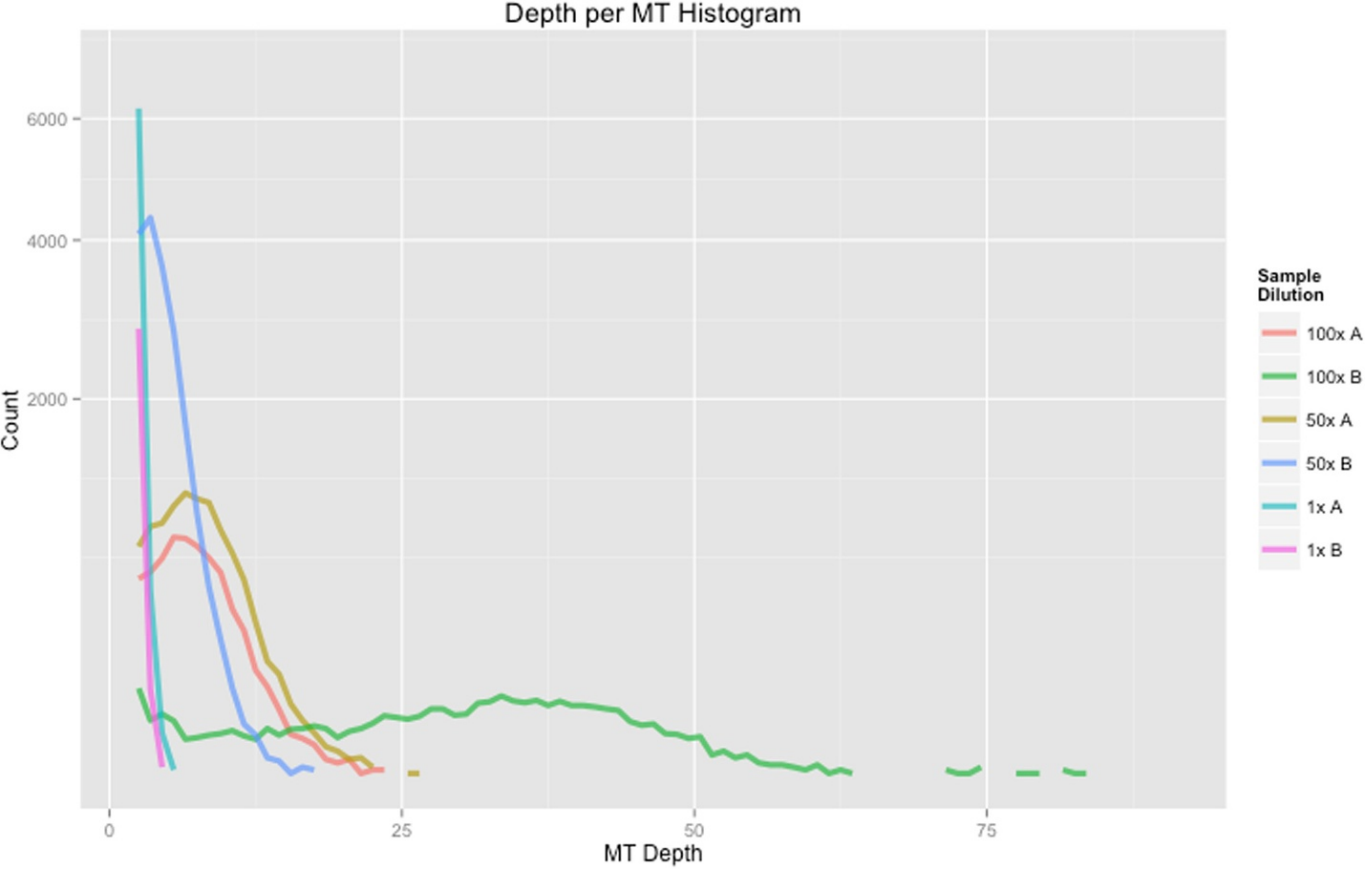
**MT depth histograms for each sample.** The number of reads in each MT category influences the accuracy of the resulting ConSeqs. MTs with higher depth are likely to generate more accurate ConSeqs. Diluting samples helps generate more MTs with higher depth at the cost of reducing the amplicon diversity.

Diluted samples result in a greater number of MTs having high depth (Figure 3); the diversity of the original amplicon population, however, is reduced. In theory, molecule tags having high depth should generate the most accurate ConSeqs by overcoming the effects of sequencing error. This creates a trade-off between creating highly accurate ConSeqs and capturing the diversity of the amplicon population. Low-complexity samples benefit from dilution because a large number of accurate ConSeqs can be created without sacrificing information about the diversity of the amplicon population. Alternatively, for high complexity samples like soil microbial communities it may be better to sacrifice ConSeq accuracy to observe a larger portion of the amplicon population.

**Figure 4:**
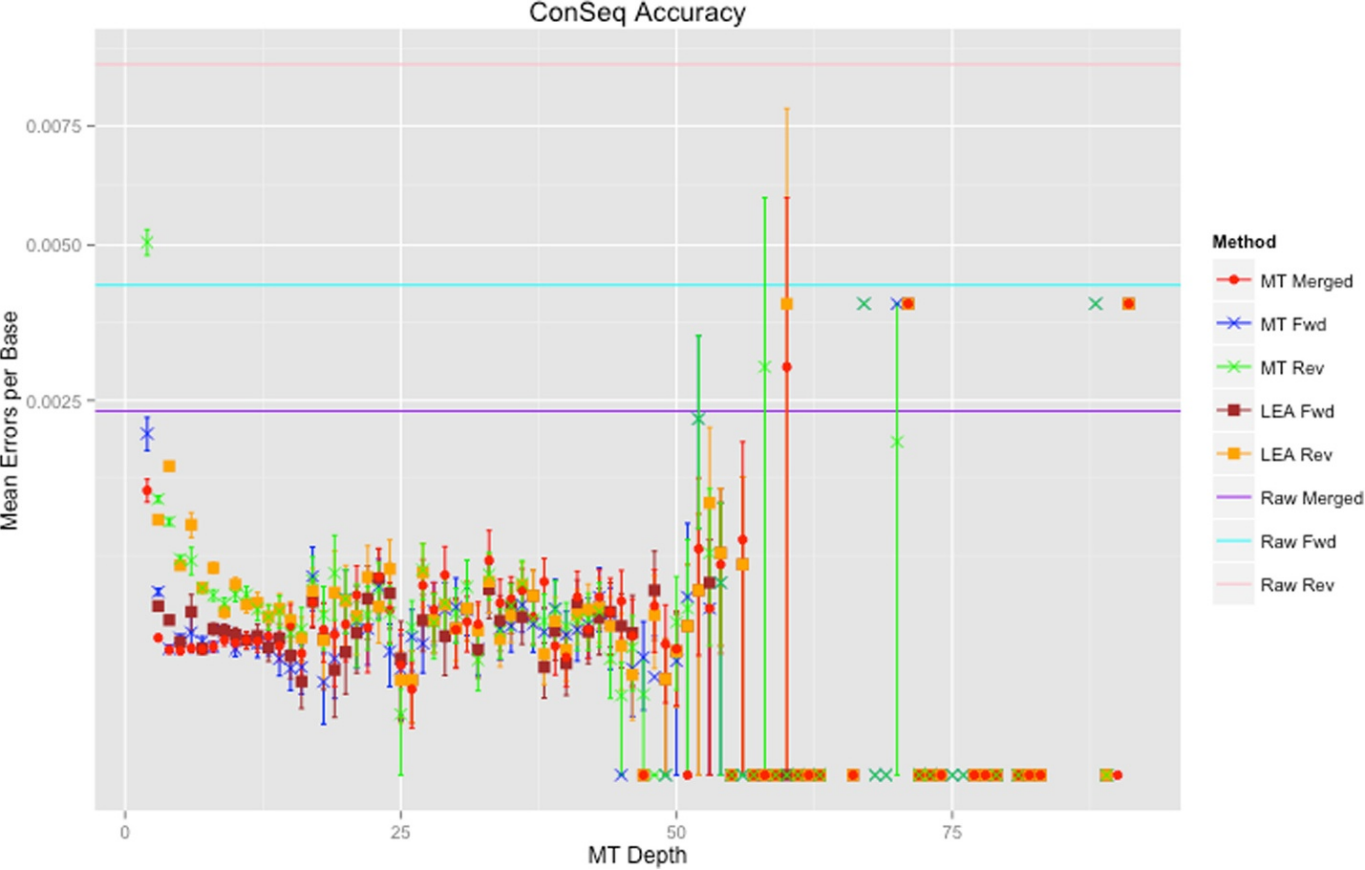
**ConSeqs error profile.** MT-Toolbox derived ConSeqs have fewer EPB than raw reads, and errors within ConSeqs are reduced as MT depth increases. MT-Toolbox ConSeqs generated from overlapping PE reads are the most accurate even at low MT depths. ConSeqs built from forward and reverse reads are slightly less accurate then overlapping PE ConSeqs. Raw reads of any type are the least accurate. MT-Toolbox ConSeqs generated from PE reads at depths ≤10 (88% of the data) are more accurate than those generated by LEA-Seq. EPB were calculated by averaging individual EPB of ConSeqs having the same depth. Error bars represent standard error and grow in length as depth increases due to fewer MTs having high depths (Figure 3).

The most accurate ConSeqs were generated from merged PE reads. Average errors per base (EPB) for ConSeqs built from ClustalW or Muscle alignments of merged PE reads was measured at ~0.00047. Without using an MSA (*i.e*. the read stacking method), ConSeqs had ~0.00112 EPB. Removing ConSeqs with a *c*-score ≤35 reduced the EPB to ~0.00089 (Additional file 1: Note S.4, Figures S7 and S8). In general, ConSeqs derived from any type of molecule tagged read were more accurate than any type of raw read (Figure 4; Additional file 1: Note S.9). Also, as depth of coverage for each MT increased, consensus sequence EPB decreased. However, for depths >15, EPB increases slightly. This trend is primarily driven by a single sample (Additional file 1: Figure S11). Why this particular sample has higher mean EPB is unclear, however it is unlikely to be caused by sample contamination or sequencing error (Additional file 1: Figure S12). In general, this outlying sample appears more error prone even at depths where other samples have very few errors (Additional file 1: Figure S11). Two examples of errors in high depth ConSeqs from this sample suggest that nucleotide misincorporation during early PCR cycles contributes to increased EPB (Additional file 1: Figure S12). Additionally, this sample has relatively fewer ConSeqs at these high depths (Figure 3) indicating that outlier ConSeqs may be inflating the mean EPB. In any case, nearly all ConSeqs in this sample still have fewer EPB than raw reads.

In general, MT-Toolbox outperforms LEA-Seq in terms of accuracy (Figure 4) and data retention. For MT depths of 2 (21% of the data), LEA-Seq failed to generate ConSeqs because it is unable resolve difference between only two reads. Alternatively, MT-Toolbox uses read quality scores to resolve such difference thereby retaining MTs of depth 2. For MT depths between 3 and 10 (67% of the data), mean EPB of MT-Toolbox ConSeqs generated from merged PE reads is lower than LEA-Seq ConSeqs. For the remaining MT depths (12% of the data) MT-Toolbox ConSeqs were either on par or slightly less accurate than LEA-Seq ConSeqs. Furthermore, because LEA-Seq was implemented to recognize only specific amplicons, a substantial number of changes to the source code were required to run LEA-Seq on these reads. This highlights the utility of MT-Toolbox where users can easily adjust parameters to build ConSeqs from virtually any amplicon or sequencing technology.

## Conclusions

Molecule tagging is a practical technique applicable to a variety of amplicon sequencing experiments, however generalizable and easily accessible software for processing custom MT data is not readily available. MT-Toolbox fills this niche by enabling efficient processing of MT data generated from a variety of protocols (Additional file 1: Note S.10). By forming ConSeqs, MT-Toolbox reduces technical errors and biases associated with PCR and sequencing thereby enabling precise measurements of DNA template abundance in mixed amplicon samples.

## Availability and requirements

-  
**Project Name: MT-Toolbox.**
-  
**Project Home Page:**https://sites.google.com/site/moleculetagtoolbox/.
-  
**Operation System(s):** Unix-based system.
-  
**Programming Language:** Perl.
-  
**Other Requirements:** Perl 5.8.8 or higher, select Perl libraries, ClustalW 2.1, MUSCLE 3.8.31, FLASH 1.2.5**,** gnuplot 4.4, an X Window System, select Unix system commands.
-  
**License:** FreeBSD.
-  
**Any restrictions to use by non-academics:** None.

## Electronic supplementary material

Additional file 1: **Supplementary Information. Note S.1.**  Building the Alignment Matrix. **Note S.2.** Single Read Categories. **Note S.3.** Optimizing ConSeq Accuracy. **Note S.4.** Filtering ‘birthday paradox’ ConSeqs Using the c-score. **Note S.5.** MT-MT_Toolbox (MeTagenomics Edition). **Note S.6.** BioUtils. **Note S.7.** Digital Normalization. **Note S.8.** Cluster Parallelization. **Note S.9.** Clonal Plasmid Accuracy. **Note S.10.** Protocols Compatible with MT-Toolbox. **Figure S1.** The implementation of molecular tags used in Lundberg *et al.* 2013. **Figure S2.** Runtime in CPU seconds of ClustalW and MUSCLE for MT categories of different depths. **Figure S3.** The length distribution of reads is very narrow. **Figure S4.** The number and types of errors seen in ConSeqs generated without using an MSA algorithm (i.e. using stacked reads) for all clonal plasmid samples. **Figure S5.** Accuracy of ConSeqs generated from ClustalW, MUSCLE, or stacked reads (i.e. no MSA) alignments. **Figure S6.** A general schematic of how five overlapping PE molecule tagged reads are used to generate highly accurate consensus sequences. **Figure S7.** c-score distributions for ConSeqs generated using different methods. **Figure S8.** The correlation between c-score and read errors. **Figure S9.** A screen shot for the GUI for the basic version of MT-Toolbox. **Figure S10.** For FASTQ file IO, BioUtils is significantly faster and scales better than BioPerl. **Figure S11.** Errors per base profile for individual samples for merged PE reads where ConSeqs are built without using an MSA. **Figure S12.** Higher errors per base in sample 100x B are unlikely to be caused by contamination or sequencing errors. (DOC 674 KB)

Additional file 2: **Source code for BioUtils-v1.0.9.** (ZIP 335 KB)

Additional file 3: **Source code for MT-MT-Toolbox-v4.1.0.** (ZIP 72 KB)

Additional file 4: **Source code for MT-Toolbox-v4.1.0** (ZIP 106 KB)

## Abbreviations

MT: Molecule tag
PE: Paired-end
SE: Single-end
MSA: Multiple sequence alignment
SRC: Single read category
GUI: Graphical user interface
OTU: Operational taxonomic unit
EPB: Errors per base
SNP: Single nucleotide polymorphism
RDP: Ribosomal Database Project
LSF: Load Sharing Facility
IUPAC: International Union of Pure and Applied Chemistry
LEA-Seq: Low Error Amplicon Sequencing.
