# Supplementary material for: MT-Toolbox: improved amplicon sequencing using molecule tags
Source: BMC Bioinformatics. 2014 Aug 22;15(1):284. doi: 10.1186/1471-2105-15-284 (PMC4153912; doi:10.1186/1471-2105-15-284)
Supplement: Supplementary file 1 — Additional file 1: Supplementary Information. Note S.1. Building the Alignment Matrix. Note S.2. Single Read Categories. Note S.3. Optimizing ConSeq Accuracy. Note S.4. Filtering ‘birthday paradox’ ConSeqs Using the c-score. Note S.5. MT-MT_Toolbox (MeTagenomics Edition). Note S.6. BioUtils. Note S.7. Digital Normalization. Note S.8. Cluster Parallelization. Note S.9. Clonal Plasmid Accuracy. Note S.10. Protocols Compatible with MT-Toolbox. Figure S1. The implementation of molecular tags used in Lundberg et al. 2013. Figure S2. Runtime in CPU seconds of ClustalW and MUSCLE for MT categories of different depths. Figure S3. The length distribution of reads is very narrow. Figure S4. The number and types of errors seen in ConSeqs generated without using an MSA algorithm (i.e. using stacked reads) for all clonal plasmid samples. Figure S5. Accuracy of ConSeqs generated from ClustalW, MUSCLE, or stacked reads (i.e. no MSA) alignments. Figure S6. A general schematic of how five overlapping PE molecule tagged reads are used to generate highly accurate consensus sequences. Figure S7. c-score distributions for ConSeqs generated using different methods. Figure S8. The correlation between c-score and read errors. Figure S9. A screen shot for the GUI for the basic version of MT-Toolbox. Figure S10. For FASTQ file IO, BioUtils is significantly faster and scales better than BioPerl. Figure S11. Errors per base profile for individual samples for merged PE reads where ConSeqs are built without using an MSA. Figure S12. Higher errors per base in sample 100x B are unlikely to be caused by contamination or sequencing errors. (DOC 674 KB) [file 12859_2014_6552_MOESM1_ESM.doc]

**Supplemental Information**

**S.1 Building the Alignment Matrix**

MTs and primer sequences are removed and only amplicon sequences are used in building MSAs. We remove primer sequences because primers can bind template despite one or more mismatches and may not be representative of the template sequence. Using the following parameters the command line version of ClustalW (Larkin *et al.*, 2007) can be used to build the MSA: -output=gde,

-outorder=input, -case=upper, -quiet , and –quicktree. The MUSCLE (Edgar, 2004) parameters for building an MSA are: -diags, -quiet.

**S.2 Single Read Categories**

In order to avoid confusion with the traditional definition of singletons in metagenomics (i.e. OTU clusters containing a single sequence), MTs containing a single raw read have been termed single read categories (SRCs) in this manuscript. This is an important distinction because it is possible to have a singleton OTU containing only one ConSeq. If this ConSeq was generated by several reads (*i.e.* has a high MT depth), it is more likely to be a low abundance microbe than an artifact of technical error. Notably, Previous manuscripts may refer to SRCs as singletons (Lundberg *et al.* 2013).

**S.3** **Optimizing ConSeqs Accuracy**

MT-Toolbox includes parameters for optimizing consensus sequence accuracy. Each consensus sequence has an attribute called MT depth—the number of raw reads used to generate that consensus. As MT depth increases consensus sequence accuracy also increases (Figure 4). MT-Toolbox users can adjust the ‘Min ConSeqs Depth’ parameter to report only ConSeqs having a MT depth greater than ‘Min ConSeqs Depth’. However, there is a trade-off between ConSeqs accuracy and the number of ConSeqs reported. For example, in template overloaded samples (in which the mean MT depth is low; i.e. 1x A and 1x B) if ‘Min ConSeqs Depth’ is set to 30 then few or no ConSeqs will be reported. While these ConSeqs will be accurate, they represent only a small sample of the original population of templates. Alternatively, if ‘Min ConSeqs Depth’ is set to two then all ConSeqs are included in the output files. To capture all possible diversity represented in the sequences, SRCs may also be included in downstream analysis with the caveat that all technical errors in SRCs necessarily remain uncorrected.

ConSeqs accuracy can also be adjusted using quality control (QC) parameters. ConSeqs and SRCs can be removed from the final FASTQ output based on minimum length, minimum average quality, and the presence of ambiguous IUPAC bases. At some small frequency, a single MT sequence will tag two different DNA templates often causing the MSA for that MT to be littered with gaps. During the consensus building step, columns having a majority of gaps are removed often resulting in a shorter than expected ConSeqs. The minimum length parameter allows the user to filter out false ConSeqs. Of course, this gap enrichment-based filtering will only work when using ConSeqs generated from an MSA and not those generated using the read stacking approach. Additionally, each consensus base is assigned a quality measure by averaging across all quality scores of the chosen base. From these values, each ConSeq is assigned an average quality score. ConSeqs with low average quality can be filtered using the minimum average quality parameter. Finally, in the event of a tie when choosing a consensus base, an IUPAC coded residue is inserted representing all the equally represented bases. Any ConSeqs having ambiguous bases can be excluded in this QC process.

**S.4 Filtering ‘*birthday paradox’* ConSeqs Using the c-score**

Due to the random nature of molecule tagging, it is predicted at some small frequency that two different templates will be tagged with the same random oligonucleotide. In probability theory this principle is commonly known as the ‘*birthday paradox’* which states that in a set of randomly chosen people, some pair of them will have the same birthday simply by chance (Sheward *et al.,* 2012). In molecule tagging, simulations of 100,000 reads show that when MT lengths are 13 bases, only ~0.1% of ConSeqs are impacted by the ‘*birthday paradox’* (Lundberg *et al.,* 2013). In theory, accurate consensus sequences can still be built for one of the templates in the affected MTs provided there is a much larger number of reads representing one of the two templates. To specifically address problematic ‘*birthday paradox’* MTs we implemented a simple algorithm to score each consensus sequence based on how closely related the reads that make up that consensus sequence are to each other. We call this metric a consensus score or c-score. Each column in the MSA is first assigned a score by multiplying the mode base percentage (i.e. mode base count / total base count) by the mode base average quality score. Subsequently, these column scores are averaged across all columns to generate the c-score. A user-defined parameter sets the minimum c-score threshold. ConSeqs below this threshold are designated as low quality during the QC process. More sophisticated approaches for identifying ‘*birthday paradox’* MTs such as sequence clustering can be implemented in the future as needed.

**S.5 MT-MT-Toolbox (MeTagenomics Edition)**

For users interested in 16S metagenome profiling, we provide a plugin for streamlining relevant 16S analysis operations. A common analysis technique in 16S profiling is to build OTUs from raw sequence data. The MT-MT-Toolbox builds OTUs from ConSeqs and, if specified, can include SRCs. OTUs are built using UPARSE, a pipeline of bash commands based on the USEARCH algorithm (Edgar, 2010). OTU representative sequences are assigned a taxonomy using the RDP Classifier (Wang *et al.*, 2007) implemented in QIIME (Caporaso *et al.*, 2010). When potential contaminant sequences (e.g. chloroplast, plastid, etc.) are provided, BLAST+ (Camacho *et al.*, 2009) is used to identify and removed OTUs that match any of the given contaminants. Along with output files provided by the basic edition, MT-MT-Toolbox also contains an OTU table where host contaminants have been removed, a corresponding FASTA file of representative sequences, and a text file with taxonomy assignments for each OTU.

**S.6 BioUtils**

To reduce speed and memory requirements, we developed BioUtils, an object-oriented Perl API for sequence analysis. BioUtils includes features for FASTA/Q file IO, storing FASTA/Q sequences, sequence QC, summarizing a set of sequences, and a simple algorithm for building a consensus sequence from an MSA. While BioUtils lacks many of the complex features of BioPerl (a common FASTA/Q processing alternative), BioUtils is significantly faster than BioPerl at basic sequence operations such as FASTA/Q file IO (Figure S4). Moreover, the simplicity of BioUtils makes it significantly more memory efficient than BioPerl. While BioUtils memory improvements were not rigorously compared to BioPerl, they can easily be observed via common Unix tools such as *top* or by simply looking at the source code. BioUtils source code is released under the BSD license and available for download at

<https://sourceforge.net/projects/bioutilsperllib/?source=directory>.

**S.7 Digital Normalization**

In general, digital normalization is a technique used to reduce a dataset such that only the necessary pieces remain for building high quality results (Brown *et al.*, 2013). In molecule tagging, digital normalization can be used to reduce runtime especially for MTs having high depth. Building MSAs for high depth MTs can be particularly time consuming (Figure S2). However, when there are many fewer MTs with depths >20, as in the example dataset, global speed improvements from digital normalization can be minimal (~5 minutes faster on the example dataset). The ‘digital norm max’ parameter allows the user to specify the maximum number of raw reads to consider when building a ConSeqs for each MT. Based on the data in Figure 1, ‘digital norm max’ could be set as low as 4 and still achieve nearly maximum accuracy for overlapping PE sequences.

**S.8 Cluster Parallelization**

MT-Toolbox can be run both in serial on a single processor or in parallel on a LSF cluster. The parallelization algorithm implemented in MT-Toolbox works by processing barcoded samples independently. For example, a sequencing run containing 96 samples is parallelized across 97 nodes—one node for each sample and one node for the parent process. Currently, MT-Toolbox parallelization can only be used on an LSF based cluster, however, by modifying a single line of code, parallelization can easily be extended to other cluster systems. Using an example dataset (Lundberg *et al.*, 2013), parallelization reduced run time from ~24.9 hours to ~52 minutes when using Clustalw to build the alignment matrix.

**S.9 Clonal Plasmid Accuracy**

To test the accuracy of ConSeqs generate by MT-Toolbox, we used six clonal plasmid samples from the Lundberg *et al.* 2013 dataset (ERS342061, ERS342062, ERS342063, ERS342109, ERS342110, ERS342111). These samples were sequenced at the University of North Carolina on the Illumina MiSeq platform using v2 chemistry. DNA was prepared using a standard Illumina Nextera P1 primer (Lundberg *et al.* 2013). Of the six samples, two were diluted 100x, two were diluted 50x, and two were not diluted. Diluting samples reduces the number of original template molecules causing more MTs to have a higher depth. Including samples with higher depths was necessary to investigate the depth required to build accurate ConSeqs. Figure 4 shows that overlapping PE reads can build accurate ConSeqs even for low depth MTs.

The overlapping PE reads used in this analysis have an expected overlap of 184 bases. Longer overlaps will generate more accurate ConSeqs by correcting more bases in the overlapping step, and shorter overlaps will be less accurate by the reciprocal reasoning.

**S.10 Protocols Compatible with MT-Toolbox**

In principle, MT-Toolbox is compatible with molecule tagged reads having strictly defined MT, primer, and amplicon sequence regions with corresponding Illumina 1.8 quality scores. Future releases may include support for other quality score formats. We tested compatibility of LEA-Seq reads by downloading a small number of LEA-Seq V4 reads from the LEA-Seq website. The available LEA-Seq reads had no quality values, so for testing purposes all bases were assigned quality values of 40. MT-Toolbox successfully built ConSeqs from this subset of LEA-Seq reads.

**Supplemental Figures**


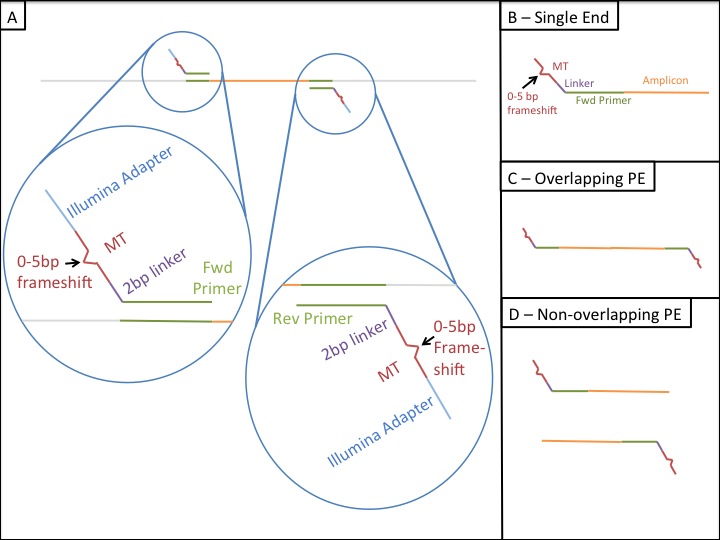


**S1**: The implementation of molecular tags used in Lundberg *et al*. 2013. A) Schematic of how the first rounds of PCR are set up. B-D) Resulting sequence patterns we expect to see in sequence output for SE, overlapping PE and non-overlapping PE reads, respectively.


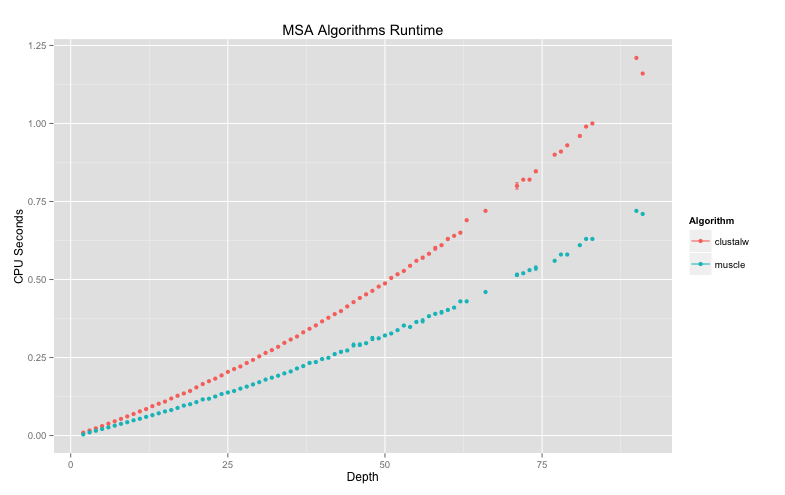


**S2**: Runtime in CPU seconds of ClustalW and MUSCLE for MT categories of different depths. MUSCLE is slightly faster. Digital normalization can be used to normalize all MTs to a user-defined depth. However, digital normalization saves only ~5 minutes when using the ClustalW algorithm on the example dataset. This suggests that the file input/output is the most time consuming step and is required a for each MT category regardless of its depth.


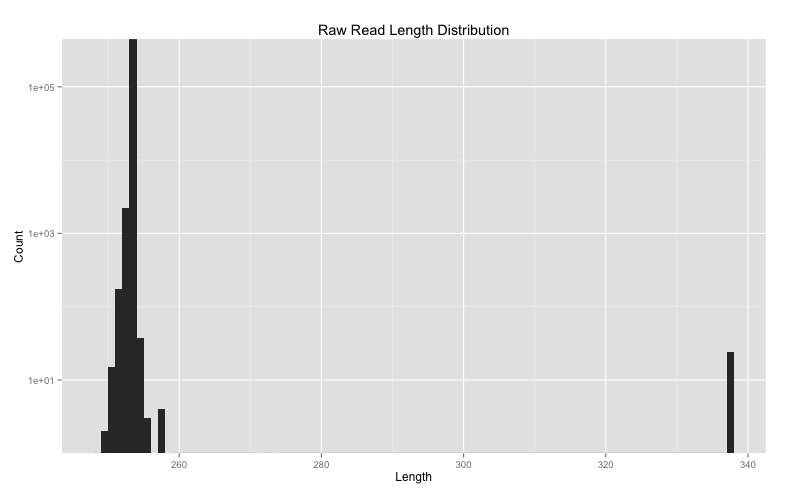


**S3**: The length distribution of reads is very narrow. Of the 449,676 clonal plasmid reads, 447,175 (99.45%) are 253bp. This suggests that the read stacking approach for building an alignment matrix is feasible.


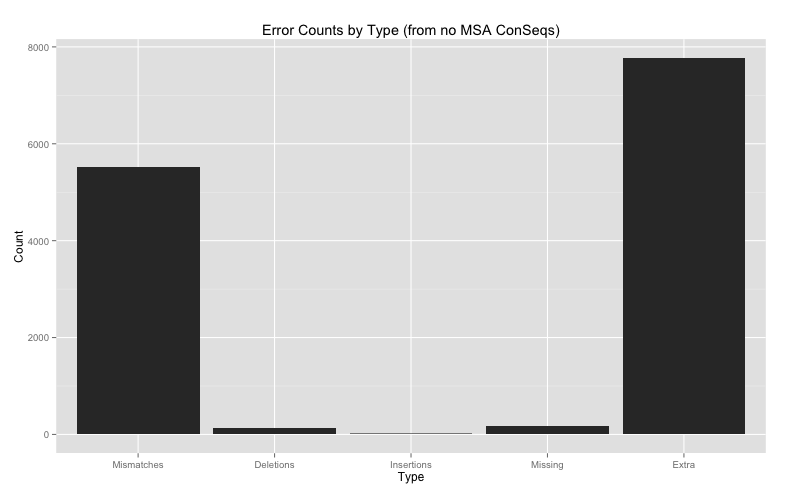


**S4**: The number and types of errors seen in ConSeqs generated without using an MSA algorithm (i.e. using stacked reads) for all clonal plasmid samples. Mismatches are much more common than deletions or insertion. Because these number were generated using BLAST output files, missing reads are most likely mismatches that were not aligned by BLAST because they fell at the beginning or end of reads. The large number of extra bases is caused by a small number of sequences where only a small portion of the ConSeq aligns to the known reference.


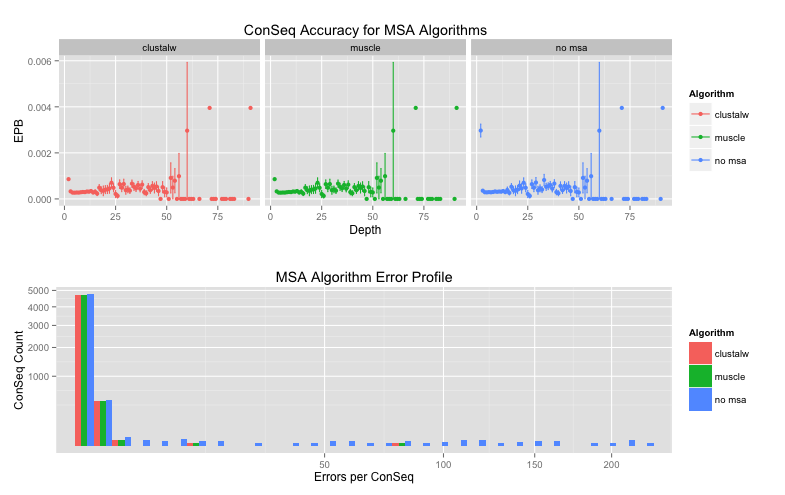


**S5**: Accuracy of ConSeqs generated from ClustalW, MUSCLE, or stacked reads (i.e. no msa) alignments. Except when MT depth is 2, stacked read ConSeqs (top-right) are equally as accurate as ClustalW (top-left) or MUSCLE (top-middle) ConSeqs. Furthermore, the errors driving the decrease accuracy of ConSeqs is likely caused by the few ConSeqs that have a large number of errors.


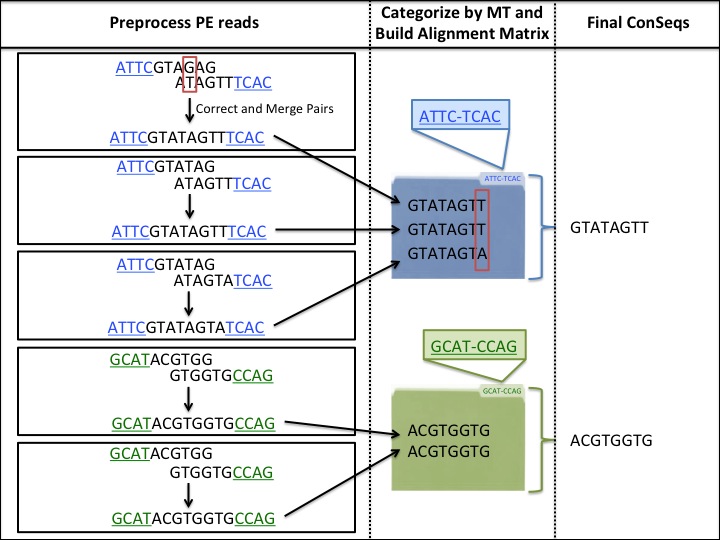


**S6**: A general schematic of how five overlapping PE molecule tagged reads are used to generate highly accurate consensus sequences. First, a preprocessing step merges overlapping PE reads using FLASH. FLASH uses read quality scores to resolve discrepancies in the overlapped portion of paired reads consequently correcting many of the sequencing errors. Second, regions (in this case only MT and amplicon) are identified using a regular expression. Third, reads are categorized by their MT. Lastly, ConSeqs are generated by building an alignment matrix and picking the most represented base at each position.


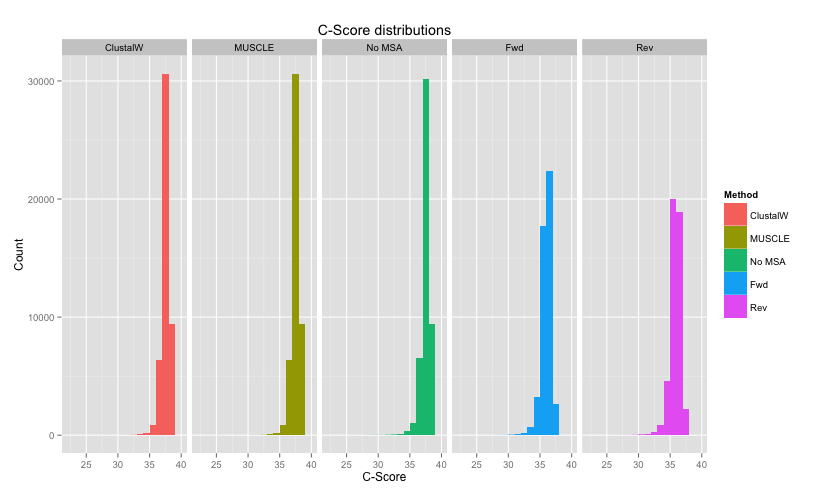


**S7**: C-score distributions for ConSeqs generated using different methods. The consensus score (c-score) can be used to remove “messy” ConSeqs potentially effected by the *‘birthday paradox’* (Note S.4) by filtering out ConSeqs with a c-score below a given threshold.

**
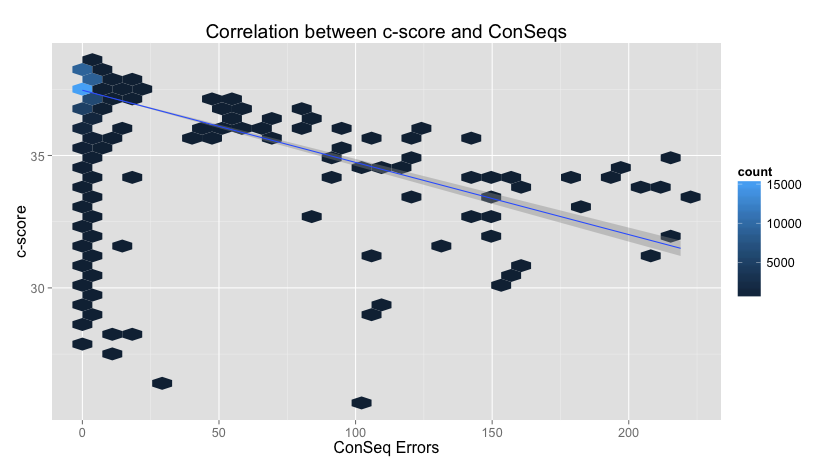
**

**S8**: The correlation between c-score and read errors. ConSeqs with more errors tend to have a lower c-score making a reasonable metric for removing low quality ConSeqs.


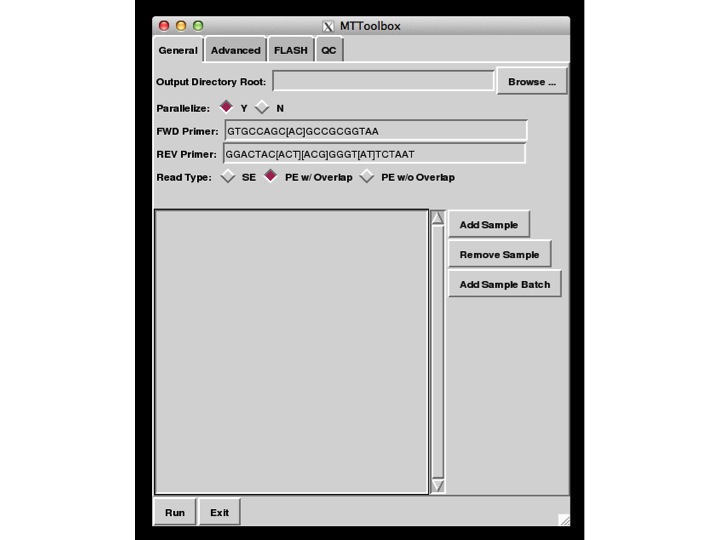


**S9**: A screen shot of the GUI for the basic version of MT-Toolbox.


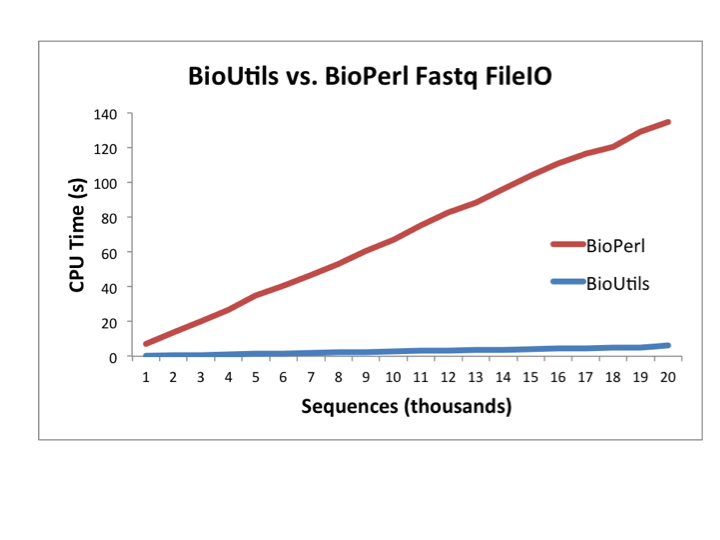


**S10**: For FASTQ file IO, BioUtils is significantly faster and scales better than BioPerl.


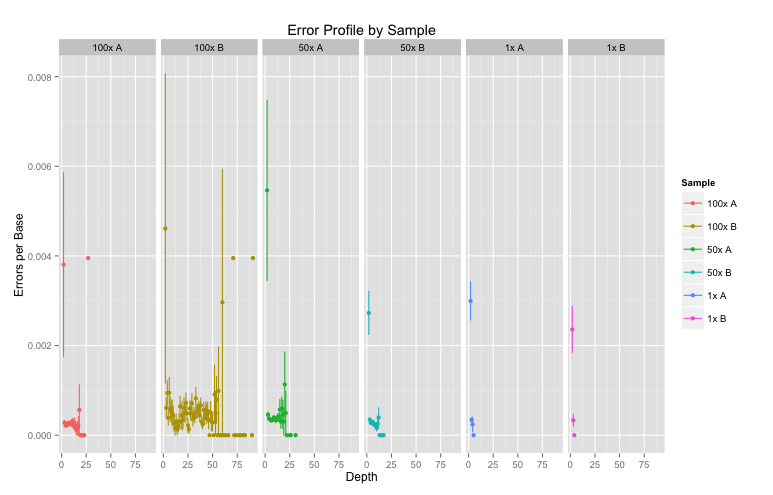


**S11**: Errors per base profile for individual samples for merged PE reads where ConSeqs are built without using a MSA. In general, diluted samples contain more MT categories with greater depths. Sample 100x B drives the high errors per base pattern seen in Figure 4 for depths >15.


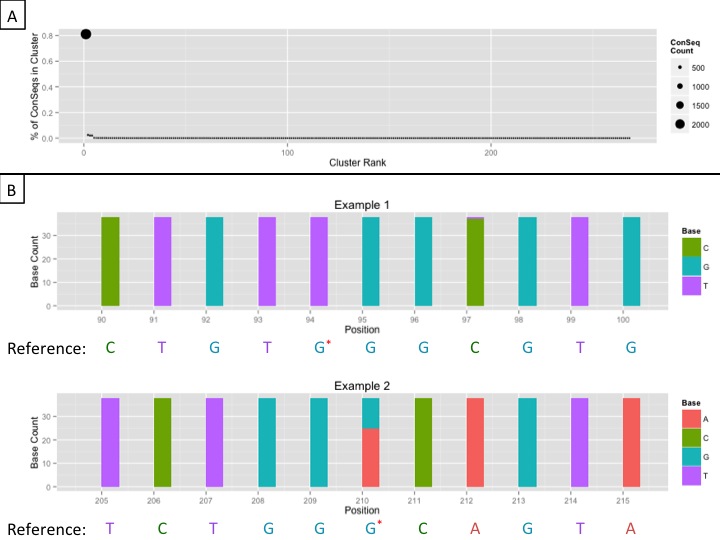


**S12**: Higher errors per base (EPB) in sample 100x B are unlikely to be cause by contamination or sequencing errors. A) All ConSeqs from sample 100x B were clustered at 100% sequence identity. Over 80% of the ConSeqs are contained in a single cluster. The next three clusters contain sequences that are exactly the same as the dominant cluster except for a single base represented as an N. Because there is a single dominant cluster and the remaining clusters contain very few sequences (>7), it is unlikely that alternative 16S contamination is the driving factor in sample 100x B’s high ERB. B) Two errors from ConSeqs having an MT depth of 38 were manually annotated. Each bar represents the number of each nucleotide base observed in each read at a given position. In example 1 the miscalled base occurs at position 94 and all 38 reads contain an incorrect base call. In example 2 the miscalled base occurs at position 210 where reads are split between the correct base, G (13 reads), and a single incorrect base, A (25 reads). Due to the consistency of incorrect base calls at these positions, sequencing errors are unlikely to be contributing to more EPB in high depth ConSeqs from sample 100x B. However, it is possible that mistakes in the early cycles of PCR amplification could lead to error patterns seen in both examples 1 and 2.
